# Identification and Functional Study of a New Missense Mutation in the Motor Head Domain of Myosin VIIA in a Family with Autosomal Dominant Hearing Impairment (DFNA11)

**DOI:** 10.1371/journal.pone.0055178

**Published:** 2013-01-29

**Authors:** Qing Sang, Xukun Yan, Huan Wang, Ruizhi Feng, Xiang Fei, Duan Ma, Qinghe Xing, Qiaoli Li, Xinzhi Zhao, Li Jin, Lin He, Huawei Li, Lei Wang

**Affiliations:** 1 State Key Laboratory of Genetic Engineering and MOE Key Laboratory of Contemporary Anthropology, School of Life Sciences, Fudan University, Shanghai, People's Republic of China; 2 Department of Otolaryngology, Eye & ENT Hospital, Fudan University, Shanghai, People's Republic of China; 3 Institutes of Biomedical Sciences, Fudan University, Shanghai, People's Republic of China; 4 Bio-X Center, Key Laboratory for the Genetics of Developmental and Neuropsychiatric Disorders, Ministry of Education, Shanghai Jiao Tong University, Shanghai, People's Republic of China; 5 Department of Otolaryngology, Chinese PLA General Hospital, Beijing, People's Republic of China; 6 Fudan-VARI Center for Genetic Epidemiology, Fudan University, Shanghai, People's Republic of China; Innsbruck Medical University, Austria

## Abstract

The *MYO7A* encodes a protein classified as an unconventional myosin. Here, we present a family with non-syndromic autosomal dominant hearing impairment that clinically resembles other previously published DFNA11 families. Affected members of the family present with an ascending audiogram affecting low and middle frequencies at young ages and then affecting all frequencies with increasing age. Genome-wide linkage analysis using Illumina Cyto-12 Chip mapped the disease locus to the DFNA11 interval in the family. A c.2003G→A (p.R668H) mutation of the *MYO7A*, is heterozygous in all affected family members and absent in 100 healthy individuals. Arg668His is located in a region of the myosin VIIA motor domain that is highly conserved among different species. Molecular modeling predicts that the conserved R668 residue plays important structural role in linking different lobes of motor domain together. In the actin-activated ATPase activity assay, the rate of NADH oxidation was higher in the wild-type myosin VIIA, indicating that the ATPase activity in the p.R668H mutant myosin VIIA was significantly destroyed.

## Introduction

Non-syndromic hereditary hearing loss is the most common sensorineural defect with both genetic and environmental origins, affecting approximately 1 out of 1000 infants. Until now, 62 genes have been identified to be associated with non-syndromic autosomal hearing impairment (http://hereditaryhearingloss.org). Myosin VIIA belongs to the Class VII unconventional myosins and consists of 49 exons; it mainly functions in the retina and inner ear. In the auditory apparatus, myosin VIIA is synthesized in the hair cells of the inner ear and participates in the formation of hair bundles associated with stereocilia [1], [2], [3]. Myosin VIIA has been implicated in Usher syndrome type 1B, atypical Usher syndrome, non-syndromic autosomal recessive hearing impairment (DFNB2) and autosomal dominant hearing impairment (DFNA11) [4], [5], [6], [7], [8]. Up to now, 340 different mutations and 248 protein variants in myosin VIIA have been reported to be associated with those diseases. (http://www.umd.be-/MYO7A/).

Until now, only seven mutations in the myosin VIIA gene have been identified as being correlated with DFNA11: p.A886_K888del in the coiled coil region [5], [9], p.G722R, p.N458I, p.A230V, p.D218N and p.G671S in the motor domain [10], [11], [12], [13] and p.R853C in the IQ motif [14].

Here, we identify a novel missense mutation in a Chinese family with progressive low and middle frequency DFNA11. The mutation p.R668H in the family is caused by a c.2003G>A transition in exon 17. We present a molecular model of the motor head domain of the human myosin VIIA protein. The p.R668H point mutation is expected to disrupt or seriously disturb the function of the motor domain. In an ATPase activity experiment, the p.R668H myosin VIIA HMM shows a lower NADH oxidation than wild-type myosin VIIA heavy meromyosin (HMM), indicating that ATPase activity is strongly disturbed in the mutant myosin VIIA.

## Materials and Methods

### Family Enrollment and Clinical Evaluations

**Figure 1:**
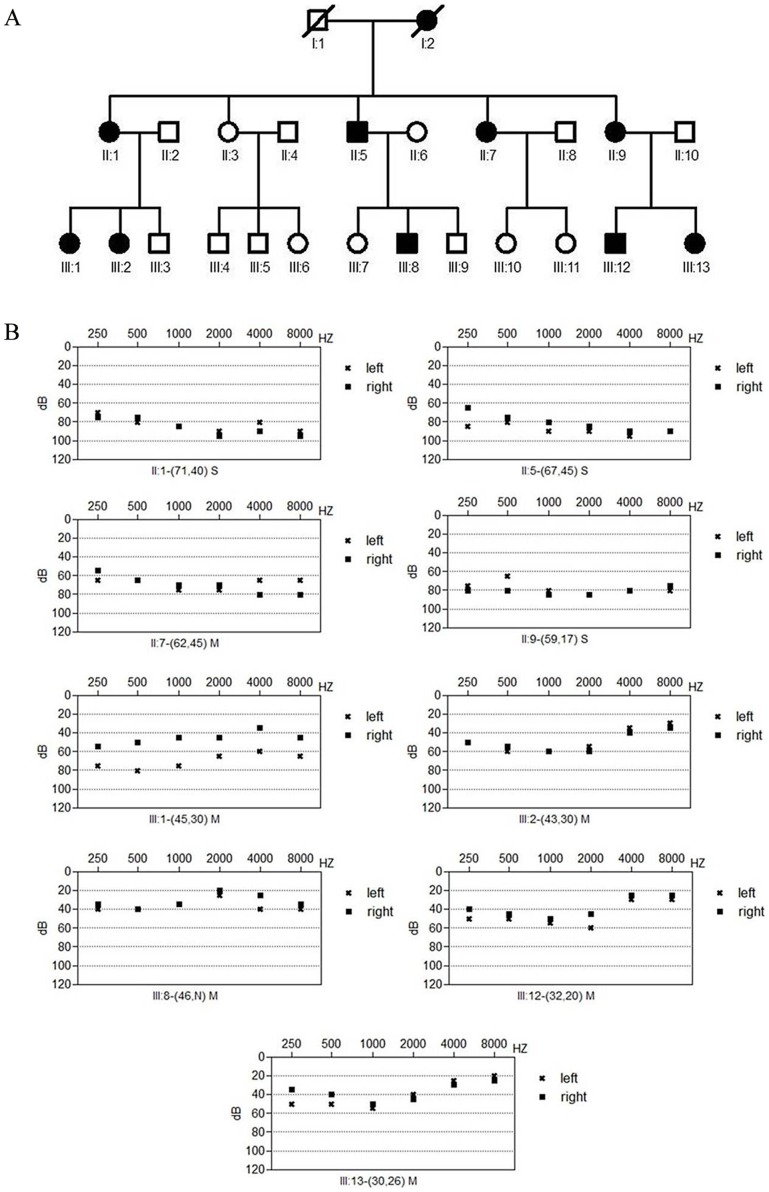
The pedigree of the Chinese family with non-syndromic hearing loss and audiogram of affected family members. (A) Pedigree of the Chinese family with non-syndromic hearing loss. Darkened symbols denote affected individuals. (B) Audiograms of nine affected members from the family. S, severe; M, moderate.

Three generations of a family with autosomal dominant late-onset progressive non-syndromic sensorineural hearing loss was recruited through Department of Otolaryngology, Eye&ENT hospital (Figure 1A). In the three generation pedigree, 23 family members participated in this study, among which 9 individuals were affected by hearing loss. Otoscopy, physical examination and pure tone audiometry (at frequencies from 250 to 8000 Hz) were performed to identify the phenotype.

Blood samples were drawn from individual II:1 to III:13. Written informed consent was obtained from all participating individuals in accordance with the ethics committee of Fudan University.

### Linkage analysis

Genomic DNA was extracted from blood collected using the genomic DNA isolation kit (Qiagen, Hilden, Germany). A genome-wide linkage analysis was performed using Illumina Cyto-12 Chip containing 300,000 SNP markers. Marker allele frequency was determined by PLINK [15]. We kept only common SNPs (MAF>10%) and generate SNPs are not in high LD with one another by PLINK [15]. A subset of 11,534 single nucleotide polymorphisms with high heterozygosity across the genome was chosen to satisfy the linkage equilibrium requirements of the Lander-Green algorithm for multipoint linkage analysis [16].. Mendelian inheritance errors were identified and removed with linkdatagen, and MERLIN was used to remove genotyping errors identified based on the inferred unlikely double recombination events between tightly linked markers [17]. A parametric linkage analysis was run assuming a fully penetrant autosomal dominant model.

### Mutational analysis of myosin VIIA

Seven mutations distributed in seven independent exons of *MYO7A* have been reported to lead to DFNA11. To confirm whether the reported mutations are causative for hearing loss in this family, seven primer pairs were designed to amplify the selected exons and exon-intron boundaries of the *MYO7A*. All the PCR products of affected and unaffected members were completely sequenced. And all the other 42 exons were also sequenced in all members (All sequencing primers were provided in Table S1). Exon 17 was also sequenced in 100 unrelated Chinese control individuals. All the PCR products were sequenced on sequencer 3730XL (Applied Biosystems) according to the standard instructions.

### Molecular modeling

To analyze the structural impact of the p.R668H missense mutation on the myosin VIIA motor domain, the sequence of the wild-type myosin VIIA motor domain was submitted to the Phyre2 server, and the 3-D structure of the myosin VIIA motor domain was modeled based on the solved motor domain structure of chick smooth muscle myosin (PDB ID: 3JO4) [18]. The generated model structure was further optimized by energy minimization using the YASARA server [19].

### Actin-activated ATPase activity assay

The heavy meromyosin (HMM) of wild-type mouse myosin VIIA cDNA and a mutant myosin VIIA HMM cDNA containing the p.R668H mutation were cloned into pFastBac1 plasmids (wild-type mouse myosin VIIA cDNA was kindly donated by Dr. Thomas Friedman from NIH). The 921 amino acid residues of HMM consists of the motor domain, five light chain-binding IQ motifs, and the coiled-coiled motif. Wild-type and mutant myosin VIIA HMM were transfected into baculovirus-Sf9 cells using lipo2000 (Invitrogen). Recombinant P0 baculovirus were collected on the third day after transfection. Then Sf9 cells were infected with P0 virus. P1 virus was collected from Sf9 cells after infection with P0 virus for 4 days. Finally, P2 virus was collected after infection with P1 virus. 500 ml suspension Sf9 cells were infected with myosin VIIA HMM P2 recombinant virus and calmodulin virus (calmodulin virus was kindly donated by Dr James Sellers at the National Heart Lung and Blood Institute). Then wild-type and mutant myosin VIIA HMM proteins were collected and purification was carried out with Ni^2+^-NTA agrose column. The concentration of the purified proteins was measured for ATPase activity assay. G-actin was extracted from the rabbit skeletal muscle using Spudish-wati method [20]. Then G-actin was homogenized into F-actin in buffer A containing 50 mM KCL and 2 mM MgCl_2_ at 4°C. Homogenized F-actin was ready for ATPase activity assay. The Actin-activated ATPase activity assay was based on a reaction in which the regeneration of hydrolyzed ATP is coupled to the oxidation of NADH. The phospho(enol)pyruvate and pyruvate kinase converts one molecule of phospho(enol)pyruvate into pyruvate when ADP is converted back into ATP. The pyruvate is subsequently converted to lactate by L-lactate dehydrogenase resulting in the oxidation of one NADH molecule. The reactions were initiated by the addition of 1 mM ATP. Wild-type and mutant mouse myosin VIIA HMM were mixed with actin in a final assay condition of 10 mM MOPS (pH 7.0), 2 mM MgCl_2_, 0.15 mM EGTA, 40 U/ml l-lactic dehydrogenase, 200 U/ml pyruvate kinase, 200 µM NADH, and 1 mM phospho(enol)pyruvate. Absorbance at 340 nm resulting from NADH oxidation was detected every one minute in 12 minutes by spectrophotometry (TECAN infinite M200) at 25°C.

### Statistical analysis

Data of the actin-activated ATPase activity assay were analyzed using Student's t-test. *P*<0.05 was considered statistically significant.

## Results

### Families and clinical evaluations

In nine affected subjects from the Chinese family, hearing impairment was symmetric, with severity varying from mild to profound on their audiograms (Table 1). The genetic defect segregating in this family shows autosomal dominant inheritance. Affected individuals had an ascending audiogram at age of onset at low or middle frequencies. The progression of hearing loss was modest in this family, affecting all frequencies with increasing age and resulting in a flat or downward sloping audiogram. Audiograms of affected members in the family are shown in Figure 1B.

**Table 1:** Clinical characteristics of the affected members in the family.

		*Age (years)*		*Hearing test PTA (dB)*		
*Subject*	*Sex*	*At testing*	*At onset*	*Left ear*	*Right ear*	*Severity of hearing loss*
II-1	F	71	40	83.7	86.2	severe
II-5	M	67	45	88.7	81.2	severe
II-7	F	62	45	70.0	71.2	moderate
II-9	F	59	17	77.5	82.5	severe
III-1	F	45	30	66.2	43.7	moderate
III-2	F	43	30	51.2	52.5	moderate
III-8	M	46	N	33.7	28.7	mild
III-12	M	32	20	47.5	41.2	moderate
III-13	F	30	26	42.5	41.2	moderate

Abbreviation: PTA, pure tone average; N, onset age was unclear.

### Linkage analysis

**Figure 2:**
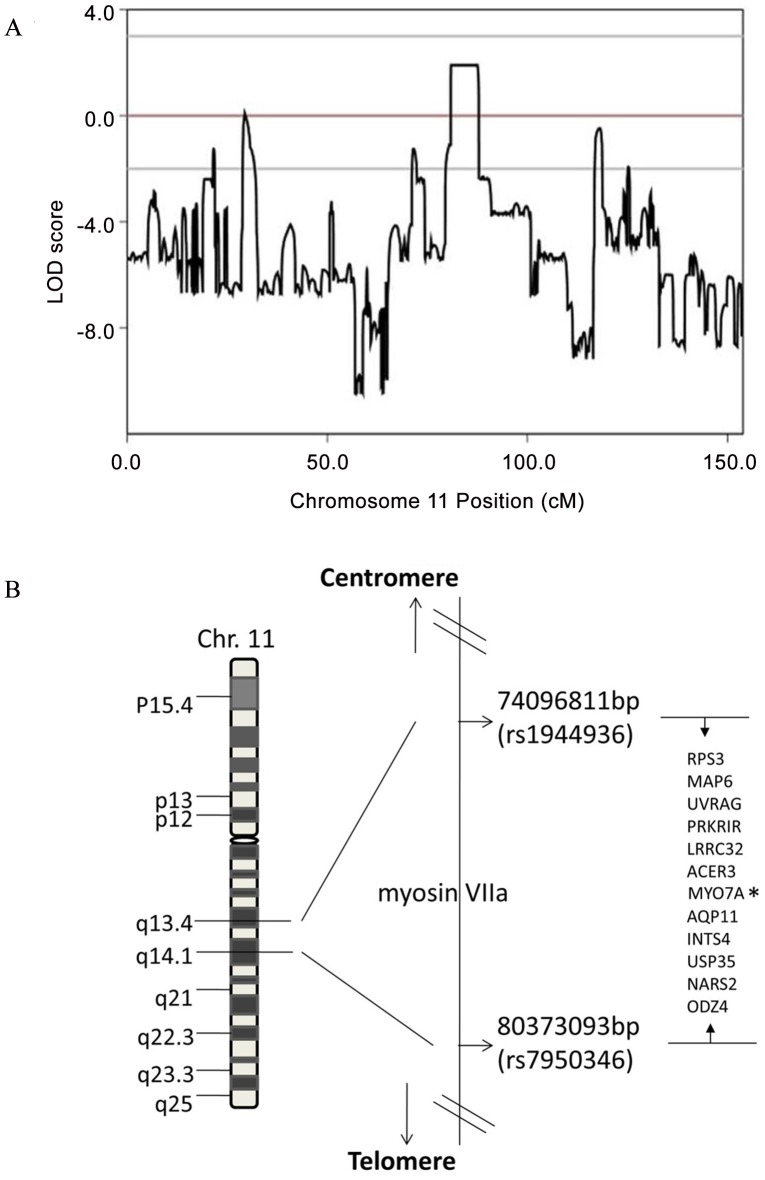
The position of the highest LOD score on chromosome 11 and schematic physical maps of 11q13.4-11q14.1. (A) Position of the identified region on chromosome 11 and its relevant LOD score. The highest score was 1.901. (B) Schematic physical and genetic maps of the 11q13.4–11q14.1 chromosomal region show the location of DFNA11. Genes that localize to the region are shown on the right.

Altogether fifteen affected and unaffected members (II:1, II:2, II:5, II:6, II:7, II:9, II:10, III:2, III:3, III:7, III:9, III:10, III:11, III:12, III:13) from the second and third generations were selected for linkage analysis. Genome-wide scanning identified an interval of 6.93 cM flanked by SNP markers rs1944936 and rs7950346 on the long arm of chromosome 11 with the maximum LOD score of 1.901 (Fig. 2A,Fig. 2B). There were 213 SNP markers with an LOD score of 1.901, indicating that the causing gene responsible for DFNA11 was located in the region 11q13.4–11q14.1 (80.922–87.90), which overlaps with the reported DFNA11 locus. As shown in figure 2A, no region in the genome reached statistical significance in this family, according to the general convention of regarding a LOD score as significant, when it reaches or exceeds 3. However, a LOD score of 1.901 appears to be close to the theoretically possible maximal LOD score in the family studied, and is a strong indicator for linkage to the DFNA11 locus. No other region in the genome showed linkage with the disease locus and >90% of the genome were excluded to harbor the disease locus by revealing a LOD score <−2.

### Mutation analysis

**Figure 3:**
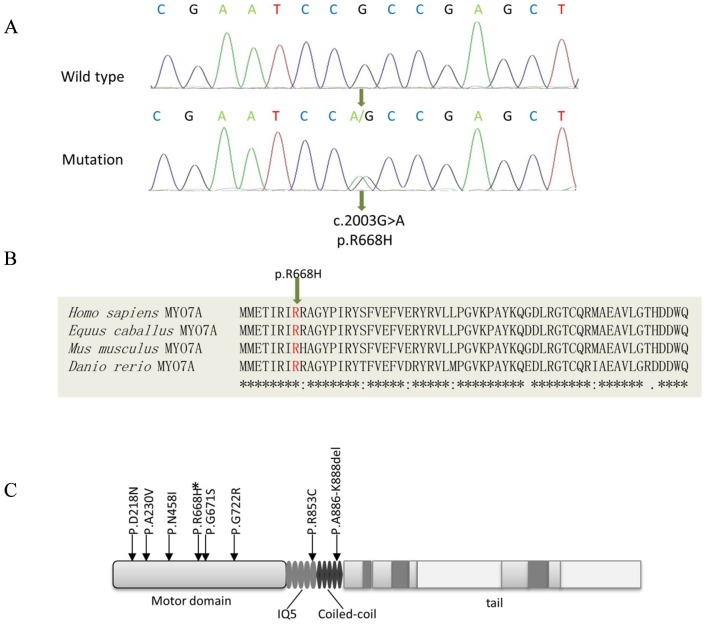
An evolutionarily conserved MYO7A head domain residue is mutated in the pedigree. (A) DNA sequence chromatograms showing heterozygous c.2003G>A (p.R668H) in affected individuals compared to homozygous R668 in controls. (B) Protein alignment of myosin VIIA in different species, which show the conservation of the residue p.R668H. (C) This schematic representation of myosin VIIA functional regions indicates the location of the eight mutations associated with DFNA11. The p.R668H mutation was marked with * above.

Using the UCSC Genome Browser (http://genome.ucsc.edu/cgi-bin/ hgGateway), we found that there are approximately 47 genes located in the region of interest. Because the *MYO7A* locus is within the interval we identified, we analyzed this gene for the presence of mutations. To date, seven mutations of myosin VIIA which cause DFNA11 have been described. Therefore, the seven exons in which these mutations are located were analyzed in second- and third-generation individuals by DNA sequencing. A novel mutation p.R668H was found in all affected family members, caused by an A to G transition at nucleotide 2003 in exon 17. The identified mutation was not reported in any public database, such as dbSNP, 1000genomes and the NHLBI project. The novel mutation p.R668H was found to be heterozygous in all affected family members, but it was absent in unaffected family members. All the other 42 coding exons were also sequenced in all affected members, but no other mutations were found. Then, we scanned exon 17 of *MYO7A* in 100 unrelated Chinese control individuals. Consequently, no nucleotide variants were detected (Fig. 3A).

### Molecular modeling

**Figure 4:**
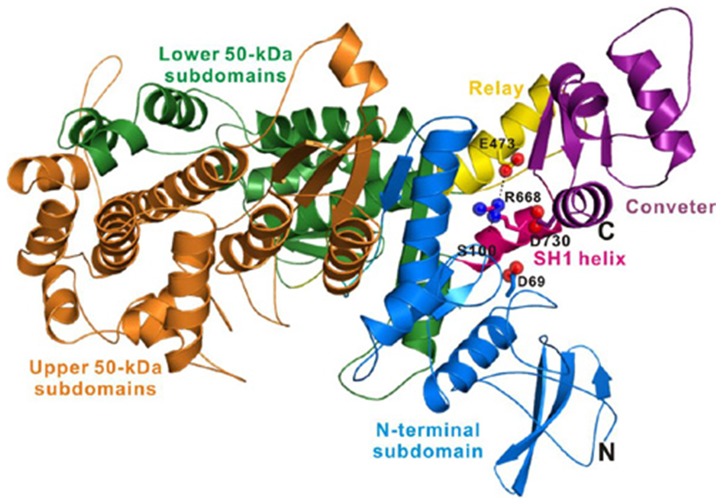
The structural analysis of the MYO7A R668H substitution. The combined ribbon and stick-sphere representation indicate the predicted structure model of the myosin VIIA motor domain. The conserved R668 residue likely plays an important structural role linking together different lobes of the motor domain. Therefore, the p.R668H point mutation is expected to disrupt or disturb the motor domain function.

The R668 residue is located in the myosin VIIA motor domain, and this residue is highly conserved based on a sequence alignment analysis of *MYO7A* from different species (Fig. 3B). Therefore, we wondered whether the identified p.R668H mutation could induce structural defects resulting in the hearing impairment found in this family. To test this hypothesis, we built a structural model based on the motor domain structure of chick smooth muscle myosin (PDB ID: 3JO4), which shares high sequence identity with the motor domain of human myosin VIIA. The R668 residue of myosin VIIA is located within the SH1 helix that connects the N-terminal subdomain and the C-terminal converter. In the structural model, the conserved R668 residue plays an important structural role in tethering together different lobes of the motor domain. R668 contributes to the formation of a pair of salt-bridges with the conserved residue E473 in the so-called relay element, as well as forming a hydrogen bond with the backbone of the S100 residue in the N-terminal subdomain (Fig. 4). The functions of the R668 residue may also play a role in forming charge-charge interactions with D69 in the N-terminal subdomain or E730 in the converter element (Fig. 4). Considering the critical role of the SH1 helix in transmitting structural changes within the motor head, the p.R668H missense mutation is expected to distort the structure and function of the SH1 helix and thereby disrupt or disturb the function of myosin VIIA motor domain.

### Actin-activated ATPase activity assay

**Figure 5:**
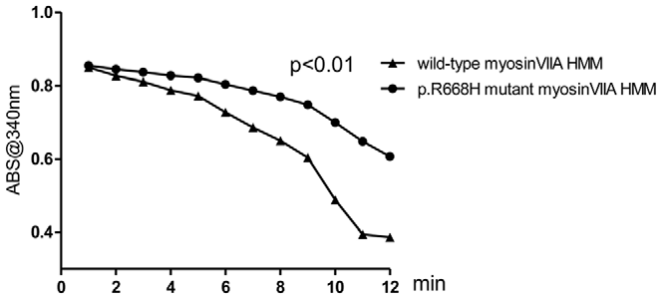
Actin-activated ATPase activity of the wild-type and mutant myosion VIIa HMM. ATPase activity analyzed by NADH oxidation. The horizontal axis represents the study period monitored. The vertical axis represents the absorbance at 340 nm.

To test the prediction that the p.R668H mutation would seriously disturb the function of the motor domain of myosin VIIA, we performed an actin-activated ATPase activity assay. Myosins are actin-binding molecular motors that use the enzymatic conversion of ATP into ADP and inorganic phosphate (Pi) to provide the energy for movement [7]. This assay is based on a reaction in which the regeneration of hydrolyzed ATP is coupled to the oxidation of NADH. The phospho(enol)pyruvate and pyruvate kinase converts one molecule of phospho(enol)pyruvate into pyruvate when ADP is converted back into ATP. The pyruvate is subsequently converted to lactate by L-lactate dehydrogenase resulting in the oxidation of one NADH molecule. The assay measures the rate of the NADH absorbance decrease at 340 nm, which is proportional to the rate of steady-state ATP hydrolysis [21]. The reactions were initiated by the addition of 1 mM ATP. We found that the rate of NADH oxidation of the mutant myosin VIIA was significantly reduced comparing with the wild-type (Fig. 5), indicating that the ATPase activity was lower in the p.R668H mutant myosin VIIA.

## Discussion

Mutations in the *MYO7A* have been reported to cause non-syndromic autosomal dominant hearing loss. Until now, only seven mutations in the *MYO7A* were known to segregate with DFNA11. Five of the seven mutations were located in the motor domain of the myosin VIIA gene, including p.G722R, p.N458I, p.A230V, p.D218N and p.G671S [10], [11], [12], [13]. In the present study, we identified an eighth mutation in a family in which progressive non-syndromic autosomal dominant hearing loss segregates with the DFNA11 locus. Through linkage analysis, we successfully mapped the causative locus to chromosome 11 at 11q13.4–q14.1. Sequence analysis revealed that an Arg668His change in the myosin VIIA gene product leads to the hearing loss observed in the family we studied. All affected family members are heterozygous for this mutated locus. The missense mutation is located in the highly conserved motor domain of the myosin VIIA gene. The R668H residue was predicted by modeling to seriously disturb the function of the motor domain of the protein. In the actin-activated ATPase activity experiment, the rate of NADH oxidation by wild-type myosin VIIA activity was higher than by mutant myosin VIIA, indicating that the ATPase activity was significantly lowered in the mutant myosin VIIA.

Myosin VIIA is a mechanoenzyme that uses actin-activated ATP turnover to power interactions with actin filaments to produce force or drive directed movements [22]. The human myosin VIIA heavy chain is composed of the N-terminal motor domain, a neck region with 5 IQ motifs, and a complex tail region. The activity of myosin VIIA is regulated by the interaction between the motor domain and the tip of the tail [23]. The myosin VIIA motor is composed of a central core and extensions to this central core form the actin-binding site and the relay, converter and the lever-arm regions. In the human cochlea, myosin VIIA is involved in hair bundle morphogenesis and mechanotransduction [1], [2], [3]. Five of the seven reported mutations are located in the motor domain, while two of these mutations are in the IQ motifs (Figure 3C).

Although molecular modeling demonstrates that all seven mutations would disable the function of myosin VIIA, only the p.R853C mutation in the IQ5 region was found to impair the calmodulin binding of myosin VIIA using an *in vivo* functional assay [14]. Using an NADH ATPase activity assay, we showed that the p.R668H mutation in the motor domain reduces the ATPase activity of myosin VIIA. When bound to ATP, the myosin VIIA head domain interacts with actin filaments to produce force- or drive-directed movements [22]. It is strongly suggested that this actin-binding ability depends on the structural stability of the myosin VIIA active site in conjunction with both the lever arm and the actin-binding interface [24]. Mutations in these sites may disrupt the link between the active site and the converter domain, in turn destroying the actin-binding efficiency of myosin VIIA. By pursuing the same activity experiment, another mutation p.R244P in the head motor of *MYO7A* was also found to seriously destroy the function of myosin VIIA [25], which further explained the genotype and phenotype in mutations of *MYO7A* and deafness. Today, the vast majority of mutations in the *MYO7A* are associated with autosomal recessive hearing loss or Usher syndrome. Generally, affected patients have inherited mutations from both parents. The nature of these mutations, which often create premature stop codons and are supposed to undergo nonsense-mediated mRNA decay and rare functional studies suggest that these recessive mutations cause disease by loss-of-function. Assuming that the p.R668H protein is produced, then the mutant protein is assumed to be trafficked to the site of action, to bind to actin but to be unable to confer normal movement to the complex or is unable to reach an appropriate confirmation due to comprised ATPase function. Spatio-temporally accumulation of dysfunction myosin VIIA molecules at actin bundles might confer a dominant negative effect on wild-type myosin molecules. This hypothesis will be addressed in future experiments.

## Supporting Information

Table S1 Sequences of primers of all 49 exons in *MYO7A*.
(DOC)
