# Supplementary material for: Identification and Functional Study of a New Missense Mutation in the Motor Head Domain of Myosin VIIA in a Family with Autosomal Dominant Hearing Impairment (DFNA11)
Source: PLoS One. 2013 Jan 29;8(1):e55178. doi: 10.1371/journal.pone.0055178 (PMC3558421; doi:10.1371/journal.pone.0055178)
Supplement: Table S1 — Sequences of primers of all 49 exons in MYO7A. (DOC) [file pone.0055178.s001.doc]

Table S1:

The primers sequences of all 49 exons in *MYO7A*.

| **Primer name Sequence (5ˊto 3＇)** | |  |
| --- | --- | --- |
| E1-F | GCAGTGGTCAGAGGGAGAGA | |
| E1-R | GGGAACAGGTTTCAGACTAG | |
| E2-F | CTGGGACTATAAGCCAGATC  GAGGTGAGAATGGGGAACCA | |
| E2-R |
| E3-F | TGCTCCAGAAGGTTGCTAGT | |
| E3-R | GCATCTGAGAGGCAGAAGTG | |
| E4-F | TGACTGAACCCTGTTAGAAC | |
| E4-R | GCACAGCGGACAAAGTCTCA | |
| E5-F | CACATGACTCCAAAGCCAGG | |
| E5-R | AGAGCTTGATGGCATTTCCG | |
| E6+E7-F | TGGGCTGAGTTCCAGTTGGT | |
| E6+E7-R | GGCTTGTGGAGAAGCCCATC | |
| E8-F | GATGGGCTTCTCCACAAGCC | |
| E8-R | CAGACAAGCCAGCAAGAAGC | |
| E9-F | TGTCAGGCAGAAAGGGCCTT | |
| E9-R | CCCAGCTTCCATCCCAAAGA | |
| E10-F | GTCACAATGCTGATGCCCTC | |
| E10-R | CCAGCAATGTGGTTCCTACA | |
| E11-F | GTGCTTAGTGGAGGCAGTGGT | |
| E11-R | ACCCAGATGTCTACCCACCTC | |
| E12-F | CGGCACTTTGTTCCACACAAGG | |
| E12-R | AGGGTCCACCGTGATGACGGAA | |
| E13-F | GGAGGTGGACTTGACAATTC | |
| E13-R | ATTCCCAAATGGCAGATGCC | |
| E14-F | GTGTAGTTCCAATTCATCCAC | |
| E14-R | ACTGAATCCAACACTTGGCCA | |
| E15-F | TCTCTATGATCTTGGGCAAG | |
| E15-R | AGCCACAGCAAAGCTCCATA | |
| E16-F | TATCTGGAGTCCAGGCTCCAG | |
| E16-R | CATGGTAGAGGCAGGATCTGA | |
| E17-F | CCACGACAGGCCTCACTTTAT | |
| E17-R | ACACCACCATGTGTGAAGTCC | |
| E18-F | CCAGCTGAGGTCACACTTCGA | |
| E18-R | AGATCCCACCTGCTCCTCCAG | |
| E19-F | TGAGTTCTTGACCTGTGCTCCC | |
| E19-R | TGCATGCCATGCATGTGTGCA | |
| E20-F | GGGCAGGCATTATTCTACAC | |
| E20-R | ACAGAGGTCCATGTGACCCAG | |
| E21-F | TAATCTGAGAGGAGACTGGG | |
| E21-R | TCCCAGGTATGTGCACTCCT | |
| E22-F | CGCTGGGTGACCTAGAGAAT | |
| E22-R | GGCTGAGTGGGTCTAGTGTG | |
| E23-F | TGGCTTGTTGAGAGGCCTCT | |
| E23-R | GACCCAGTTCATCTCTGCTC | |
| E24-F | GAGCAGAGATGAACTGGGTC | |
| E24-R | CGCAGTGAAGACTATAGTCAG | |
| E25-F | CTGACTATAGTCTTCACTGCG | |
| E25-R | GTCGGTGAGGCAGAGATGAA | |
| E26-F | TTCATCTCTGCCTCACCGAC | |
| E26-R | CTGAGGACAGACAGTGTCCA | |
| E27-F | GTAATGACAGTGATGGGGAG | |
| E27-R | CAAGGCAAGACCCTCCAGCA | |
| E28-F | GATGATCCTGTCTCCAAAGCC | |
| E28-R | AAAGAGGTGGCTGCCTGAGA | |
| E29-F | GACGTGGCTGGAAATAGATG | |
| E29-R | GTAAGGCCAGCAAATGGAGTC | |
| E30-F | CACAGAAAGCAGAGAGCCAAAG | |
| E30-R | AGGTGCTGAAGAGCACTCAGCA | |
| E31-F | GCTAGAATCTGGTCTGCCTCC | |
| E31-R | GTGTCCATCCAGGAGCCTGAA | |
| E32+E33-F | GTCACAGTGATGCACTTCCCT | |
| E32+E33-R | GCAAGGTTAAGTCTGATTCCC | |
| E34-F | CGAATGAGAAAGGTGGGGAC | |
| E34-R | GGTACCTGAGAATTTGTAGGC | |
| E35-F | TGCTCTCTGAGCCTCAGTTTCC | |
| E35-R | ACTGCCCAATCACGTGCAACCA | |
| E36-F | GACAGGCAAAGGAGAAGTAG | |
| E36-R | GCCAGCACCATGCTTCTTGTA | |
| E37-F | GAGAGTTGTGACAAGGTGGAG | |
| E37-R | AGATCCCAAGATCATCTTCAC | |
| E38-F | CTCAGCACAAAGGAGGCAGC | |
| E38-R | GGGTCATCCTGGCAGACAGA | |
| E39-F | TCACATGCAGGGCAGGGAAGA | |
| E39-R | GAGGCAGCCCCTTGCATATCA | |
| E40-F | TTGTGAGTGTGCAGCCTGAGG | |
| E40-R | GAAGGACTGCATCCAGGCAGA | |
| E41-F | CTGAGATAGGGTAAAGGTCAG | |
| E41-R | ACCTCCGTCTAGCTGGCAGTT | |
| E42-F | TACAGATGAGGAGCTCAAGG | |
| E42-R | AGTCGATGGCCTTCGTCCTG | |
| E43-F | CAAGGACCAAAGACTTCCTC | |
| E43-R | AGTTCCTCTCTCTTCCGTCTG | |
| E44-F | GAGTGGAGTCTTCCCTGAGAAG | |
| E44-R | TCCCTGCACAGAGGAGTGCTCA | |
| E45-F | TGAGCACTCCTCTGTGCAGGGA | |
| E45-R | CCAGTGTCCTCTCTGCCACA | |
| E46+E47-F | CAGACTGAGAGGCTCTTCCAG | |
| E46+E47-R | GCCTCATCAAGTACAAGAACC | |
| E48-F1 | GAGGACTCTGAGCTCTGAGCT | |
| E48-R1 | CTTGCCAAGCTGCTGCTCTC | |
| E48-F2 | CAGGTCTGGGAAGGAAATCC | |
| E48-R2 | GAGTCTAGAGATAGATGGTGGC | |
| E49-F | GTCCAAACCAAGGTGTGGAG | |
| E49-R | CAGACCTAAGCTGAGCCTCA | |
